# Development of Functional Genomic Tools in Trematodes: RNA Interference and Luciferase Reporter Gene Activity in *Fasciola hepatica*

**DOI:** 10.1371/journal.pntd.0000260

**Published:** 2008-07-09

**Authors:** Gabriel Rinaldi, Maria E. Morales, Martín Cancela, Estela Castillo, Paul J. Brindley, José F. Tort

**Affiliations:** 1 Department of Tropical Medicine, Tulane University Health Sciences Center, New Orleans, Louisiana, United States of America; 2 Departamento de Genética, Facultad de Medicina, Universidad de la República, Udelar, Montevideo, Uruguay; 3 Sección Bioquímica, Facultad de Ciencias, Universidad de la República, Udelar, Montevideo, Uruguay; Queensland Institute of Medical Research, Australia

## Abstract

The growing availability of sequence information from diverse parasites through genomic and transcriptomic projects offer new opportunities for the identification of key mediators in the parasite–host interaction. Functional genomics approaches and methods for the manipulation of genes are essential tools for deciphering the roles of genes and to identify new intervention targets in parasites. Exciting advances in functional genomics for parasitic helminths are starting to occur, with transgene expression and RNA interference (RNAi) reported in several species of nematodes, but the area is still in its infancy in flatworms, with reports in just three species. While advancing in model organisms, there is a need to rapidly extend these technologies to other parasites responsible for several chronic diseases of humans and cattle. In order to extend these approaches to less well studied parasitic worms, we developed a test method for the presence of a viable RNAi pathway by silencing the exogenous reporter gene, firefly luciferase (fLUC). We established the method in the human blood fluke *Schistosoma mansoni* and then confirmed its utility in the liver fluke *Fasciola hepatica*. We transformed newly excysted juveniles of *F. hepatica* by electroporation with mRNA of fLUC and three hours later were able to detect luciferase enzyme activity, concentrated mainly in the digestive ceca. Subsequently, we tested the presence of an active RNAi pathway in *F. hepatica* by knocking down the exogenous luciferase activity by introduction into the transformed parasites of double-stranded RNA (dsRNA) specific for fLUC. In addition, we tested the RNAi pathway targeting an endogenous *F. hepatica* gene encoding leucine aminopeptidase (*Fh*LAP), and observed a significant reduction in specific mRNA levels. In summary, these studies demonstrated the utility of RNAi targeting reporter fLUC as a reporter gene assay to establish the presence of an intact RNAi pathway in helminth parasites. These could facilitate the study of gene function and the identification of relevant targets for intervention in organisms that are by other means intractable. More specifically, these results open new perspectives for functional genomics of *F. hepatica*, which hopefully can lead to the development of new interventions for fascioliasis.

## Author Summary

Reverse genetics tools allow assessing the function of unknown genes. Their application for the study of neglected infectious diseases could lead eventually to the identification of relevant gene products to be used in diagnosis, or as drug targets or immunization candidates. Being technically more simple and less demanding than other reverse genetics tools such as transgenesis or knockouts, the suppression of gene activity mediated by double-stranded RNA has emerged as a powerful tool for the analysis of gene function. RNAi appeared as an obvious alternative to apply in complex biological systems where information is still scarce, a situation common to several infectious and parasitic diseases. However, several technical or practical difficulties have hampered the development of this technique in parasites to the expectations originally generated. We developed a simple method to test the presence of a viable RNAi pathway by silencing an exogenous reporter gene. The method was tested in *F. hepatica*, describing the conditions for transfection and confirming the existence of a viable RNAi pathway in this parasite. The experimental design created can be useful as a first approach in organisms where genetic analysis is still unavailable, providing a tool to unravel gene function and probably advancing new candidates relevant in pathobiology, prevention or treatment.

## Introduction

Parasitic diseases are a major problem worldwide being not only a health issue but also, when affecting productive species, an important factor in the economy. Advances in biochemistry and molecular biology of parasites have made possible to identify at the molecular level several candidate mediators in the parasite-host interaction. However, the validation of the role of these molecules is hampered in many cases by the absence of appropriate tools of analysis, in particular functional genomics approaches to address the importance of a target gene in the pathogen. While functional genomics of parasitic nematodes have benefited from the advancements in the model species *Caenorhabditis elegans*, in parasitic flatworms, this avenue has been explored almost exclusively in species of the genus *Schistosoma*, but the techniques are still in their infancy (reviewed in [1]–[4]). Transfection with reporter genes using endogenous promoters has been attempted successfully in adults, miracidia and sporocysts of *S. mansoni* by biolistic approaches [5]–[11]. Transient transfection by electroporation was achieved in schistosomules and adults of *S. mansoni* [12] and *S. japonicum* [13]. More recently advancements towards stable transduction using retroviral and transposon retroposon sequences have been made [14],[15]. Gene silencing by RNA interference was first demonstrated in schistosomules by soaking worms in double stranded RNA [16] and later extended to sporocysts [17]. Electroporation was also tested as a delivery method for dsRNA providing for stable and long term effects [18]. These initial reports paved the ground for the use of the methodology in the analysis of gene function [19]–[23]. Although the possibilities of this techniques in parasitic flatworms are still far away from the systematic use of RNAi at genomic scale as initiated in free living planarians [24]–[27], the existence of extensive genomic and expression information for *S. mansoni* and *S. japonicum* [28]–[32] provide opportunities for advances in the discovery of new anti-schistosome interventions.

Apart from schistosomes, reverse genetics techniques have been reported in just three other species of parasitic flatworms, but they are prominent examples of the relevance of these techniques for parasite biology and control. Germinal cells from the cestode *Echinococcus multilocularis* were successfully maintained in culture and transfected with reporter plasmids. Furthermore, these cells were also experimentally infected with *Listeria monocytogenes*, a facultative intracellular bacteria used as DNA delivery system for genetic manipulation of mammalian cells [33]. Germ cells development in the monogenean *Neobenedenia girellae* was altered by soaking with dsRNA of two *vasa* related genes, opening promising avenues for control measures [34]. Also, the role of cysteine proteases in parasitic invasion was validated in an assay using RNAi directed against cathepsins L and B in the trematode *Fasciola hepatica* [35]. We are interested in extending these methodologies to other parasitic flatworms, and to gain insights from comparative analysis of genes in less comprehensively studied flukes. For this purpose, we attempted to build on the growing schistosome reverse genetics background, in order to generate a simple reporter gene assay to be tested in other helminthes.

While schistosome infection is the most relevant trematode infection of humans worldwide, fascioliasis holds a similar status in ruminants, with at least 700 million animals infected, and at least one fourth of the world livestock grazing at areas where the parasite is present. The economic losses caused by the infection are conservatively estimated at USD 3.2 billion per annum, with a more pronounced impact to rural agricultural communities in developing countries [36]. Recently, the disease has also emerged as a major zoonosis mainly in rural areas of central South America, Northern Africa and Central Asia, with approximately 2.4 million infected people worldwide and 180 millions at risk [37],[38]. Although the anthelmintic triclabendazole is effective for controlling *Fasciola* infection [39], reports on drug resistance are increasing, indicating that selection of resistant parasites may eventually compromise its use (reviewed in [40]). Genomic approaches have begun in *Fasciola*, with a small set of ESTs available (ftp://ftp.sanger.ac.uk/pub/pathogens/Fasciola/hepatica/ESTs/), and proteomics had also been applied in the analysis of parasite excreted/secreted products [41],[42] . However, despite this growing catalogue of sequences, the functional analysis and characterization of potential intervention targets is hampered by the lack of reliable methods of reverse genetics. *F. hepatica* represents a relevant candidate to evaluate the amenability of transferring reverse genetics techniques already applied in schistosomes [2],[20], particularly the suppression of gene activity mediated by double stranded RNA. At the same time that this work was under way, other colleagues demonstrated for the first time the existence of a viable RNAi pathway in this trematode [35].

In this report, we established a model test system in *S. mansoni* to indicate the presence of a viable RNAi pathway, based on the inactivation of an exogenous reporter. We transferred transfection technologies tractable in *S. mansoni* to *F. hepatica* juveniles which allowed not only an effective electroporation-based delivery method for nucleic acids to the liver flukes but also provided a method of quantitative analysis of a powerful reporter expression system. In addition, we confirmed the existence of a functional RNAi pathway in *Fasciola* using both the exogenous luciferase reporter and an endogenous protease gene. Finally, these methods should also find utility in investigation of other less characterized helminth parasites.

## Materials and Methods

### Schistosomes, *Fasciola hepatica*, parasite cultures

*Biomphalaria glabrata* snails infected with the NMRI (Puerto Rican) strain of *Schistosoma mansoni* were supplied by Dr. Fred Lewis, Biomedical Research Institute (Rockville, MD, USA). Cercariae released from infected B. glabrata snails were concentrated by centrifugation (2000 rpm/10 min), washed once with somule wash medium, and mechanically transformed by shearing off the tails by 20 passes through 22G emulsifying needles. Schistosomule bodies were isolated from free tails by Percoll gradient centrifugation [43], washed three times in wash medium and cultured at 37°C under 5% CO2 in modified Basch's medium supplemented with washed human erythrocytes [44]. Culture media were changed every second day, and the viability of the schistosomules was monitored under the microscope.

Metacercariae of *Fasciola hepatica* were purchased from Baldwin Aquatics Inc. (Monmouth, Oregon). The *in vitro* excystment was performed as described [45] with minor modifications. Briefly, metacercariae were placed in a 100 µm filter and incubated 5 min at room temperature with 1% sodium hypochlorite to remove the outer cyst wall. After an exhaustive wash in PBS, the metacercariae were incubated at 39°C in activation medium (25 mM HCl and 16,5 mM L-cysteine, 0.1% sodium taurocholate, 60 mM NaHCO_3_, 70 mM NaCl pH 8.0), and the excystment process was monitored under the microscope. After 90–180 min of incubation, newly excysted juveniles (NEJs) began to emerge, and were collected, washed several times with RPMI-1640, transferred into 6 wells plates and keep in culture at 37°C under 5% CO_2_ with Basch's medium or schistosomule wash medium (RPMI 1640 supplemented with 200 U/ml Penicillin G sulfate, 200 µg/ml streptomycin sulfate, 500 ng/ml amphotericin B, 10 mM HEPES). For longer term culture up to days, larval flukes were incubated in Basch's medium [44], culture media were replaced every fourth day and viability of NEJs monitored microscopically.

### Synthesis of mRNA and dsRNA

To synthesize firefly luciferase mRNAs (mLuc), DNA template were prepared by PCR from pGL3-Basic (Promega, Madison, WI, USA) templates as described [12]. *In vitro* transcriptions of capped RNAs from PCR DNA templates were accomplished using the mMessage mMachine T7 Ultra kit (Ambion, Austin, TX, USA) according to the manufacturer's instructions. Subsequently, LiCl-precipitated RNAs were dissolved in nuclease-free water and quantified by spectrophotometer (ND-1000, NanoDrop Technologies, Wilmington, DE).

All of the dsRNA used in the experiments were generated by *in vitro* transcription using as templates PCR products generated with gene specific primers tailed with the T7 promoter sequence. A luciferase dsRNA template encoding the full length 1,672 kb was generated using the pGL3-basic plasmid (Promega, Madison, WI) as template (F: 5′-*TAA TAC GAC TCA CTA TAG GG* T GCG CCC GCG AAC GAC ATT TA-3′; R: 5′-*TAA TAC GAC TCA CTA TAG GGG* CAA CCG CTT CCC CGA CTT CCT TA-3′). A 808 bp fragment of the *E. coli* malE gene included in the control plasmid LITMUS 28iMal from the HiScribe RNAi Transcription kit (New England BioLabs, Ipswich, MA) was amplified by PCR from the opposing T7 promoters of the vector with a T7 promoter-specific primer, generating a template for the double-stranded MalE (dsMalE) control. 482 bp from the 5′ portion of the *Fasciola hepatica* leucine aminopeptidase gene (*Fh*LAP, GenBank accession AY64459) was generated by PCR from the full length *Fh*LAP cDNA [46] using gene-targeted primers containing T7 promoter sequence F: 5′- *TAA TAC GAC TCA CTA TAG GG* ATC TGC TAC TCA ATG CTC TG-3′ and R: 5′- *TAA TAC GAC TCA CTA TAG GG*CAC TCC GTT CGC CTT GAT GT-3′ (spanning coding DNA position 389–871).

dsRNA was synthesized and purified using the Megascript RNAi kit (Ambion) according to the manufacturer's instructions. Integrity of the dsRNAs was verified by non-denaturing 1% agarose gel electrophoresis and purity was accessed by the ratio *A*_260_/*A*_280_. dsRNA was precipitated with 1 volume of 5 M ammonium acetate and 2.5 volumes of 95% ethanol after which the RNA pellet was dissolved in water. Concentration of dsRNA was determined spectrophotometrically (ND-1000, NanoDrop Technologies, Wilmington, DE).

### Electroporation of parasites

Schistosomules of *Schistosoma mansoni* or NEJs of *Fasciola hepatica* removed from culture at different time points after cercarial transformation or excystment respectively, were transformed with nucleic acids preparation as described [12]. In brief, 1,500–2,000 NEJs or 10,000 schistosomules resuspended in 100 µl of wash medium containing 5 µg of luciferase mRNA (final concentration 50ng/µl) in 4 mm gap cuvettes were subjected to square wave electroporation (125 V, 20 ms) in a BTX ElectroSquarePorator™ ECM830 (BTX, San Diego, CA). Immediately after electroporation, the larval flukes were transferred to prewarmed Basch's Medium and maintained in culture as indicated.

Fluorescent siRNA molecules (Silencer Cy 3-Labeled Negative Control #1 siRNA, Ambion, Austin, TX, USA) were used for transfection, following the same electroporation protocol describe above. Parasites were electroporated with 0, 50 or 100 ng/µl of fluorescent siRNA, respectively. Three hours after electroporation, worms were rinsed in fresh culture medium, observed in a Olympus IX81 fluorescent microscope and photographed with an Hamamatsu C4742-8012AG. Digital Camera. The parasites were maintained in culture and fluorescence detected and registered 24 hours after treatment.

For dsRNA electroporation the same protocol was followed using 300 ng/µl of dsRNA, in a final volume of 100 µl of somule wash medium. Ten µg of a plasmid DNA construct encoding luciferase driven by the actin 1.1 gene promoter of *S. mansoni* (pLuc) (from ref [14]) was used to transform schistosomules pretreated with or without luciferase dsRNA, following the same electroporation settings described above. Since there were abundant previous data on electroporation, reporter systems and RNAi in *S. mansoni* we conducted several single experiments testing different timings for the electroporation. All the results obtained were consistent within them and with previously published data. All the experiments involving *F. hepatica* juveniles were repeated three times.

### Luciferase activity assay

Parasites were harvested at different hours after electroporation, washed three times with schistosomule wash medium and stored as wet pellets at −80°C. Luciferase activity in extracts of these parasites was monitored using Promega's luciferase assay reagent system and a Sirius luminometer (Berthold, Pforzheim,Germany) [12]. In brief, pellets of parasites were subjected to sonication (3×5s bursts, output cycle 4, Heat Systems-Ultrasonics, Plainview, NY, USA) in 250 µl CCLR lysis buffer (Promega). Aliquots of 100 µl of sonicate were injected into100 µl luciferin substrate (Promega) at room temperature, mixed, and the relative light units (RLUs) were determined in the luminometer 10 s later. Duplicate samples were measured, with results presented as the average of the readings per mg of soluble fluke protein. The protein concentration in the soluble fraction of the extract was determined using the bicinchoninic acid assay (BCA kit, Pierce, Rockford, IL). Recombinant luciferase (Promega) was included as a positive control.

### Whole-mount in situ hybridization

A digoxigenin labeled antisense Firefly Luciferase riboprobe was generated by transcription according to the manufacture's protocol (Roche Applied Science) using as template a 553pb PCR product amplified from the pGL3-basic plasmid (Promega, Madison, WI) with a gene specific forward primer (F: 5′-GTG CCA GAG TCC TTC GATAG-3′) and a gene specific reverse primer tailed with the T7 promoter sequence (R: 5′- *TAA TAC GAC TCA CTA TAG GG* ACA ACT TTA CCG ACC GCG CC-3′).

Worms were electroporated with 0 (“mock control”), or 50 ng/µl of luciferase mRNA (mLuc) and fixed three hours after treatment at 4°C overnight in 4%paraformaldehyde in PBS, washed in PBS, dehydrated and stored at −20°C in 100% ethanol. After rehydration with 75%, 50% and 25% ethanol in Holfreter buffer (NaCl 2.188 g, KCl 0.031 g, CaCl_2_ 0.063 g, NaHCO_3_ 0.125 g, in 1000 ml H_2_O), the parasites were washed in PBS-T (PBS with 0.1% Triton X-100) 30 mins, and treated with proteinase K (20 µg/ml in PBS-T) for 5 minutes at 37°C. Digestion was stopped with cold Holfreter, and worms were postfixed in 4% formalin in Holfreter for 60 minutes, rinsed in Holfreter two times (20 mins each), and prehybridized for 120 minutes at 55°C in hybridization solution (50% formamide, 5× SSC, 1 mg/ml yeast tRNA, 100 µg/ml heparin, 0.1% Tween-20).

The riboprobe was desnaturalized by heating to 70°C for 3 minutes, immediately transferred to ice for 5 min, diluted to 1 ng/µl in hybridization solution and added to samples for hybridization at 55°C for 16–36 hours. After hybridization, worms were washed in 50% formamide, 5× SSC, and 0.1% Tween 20, for 60 mins, four times at 55°C, rinsed twice in MAB-T (Triton-X 100 0.1% in MAB (maleic acid 11.6 g, NaCl 9.76 g, NaOH 2N 95 ml, in 1000 ml H_2_O, pH 7,5), and then incubated for 30 minutes at room temperature in blocking solution (0.5% Roche blocking reagent in MAB-T, 5%). After blocking, parasites were incubated overnight at room temperature with 1∶2000 alkaline-phosphatase (AP)-conjugated anti-digoxigenin antibody (DIG Nucleic Acid Detection KIT- Roche Applied Science) in MAB-T, rinsed four times in MAB-T (5 min the first wash and 60 min the remaining washes), and once in AP buffer (100 mM Tris pH 9.5, 150 mM NaCl, 25 mM MgCl_2_, Triton X-100 0.1%) for 5 min. Signal was detected following incubation of the organisms in NBT- BCIP stock solution (Roche Applied Science) 5 mM levamisol in buffer AP in dark. When the chromogenic reaction was complete (2 to 4 hours), the organisms were washed twice in PBS 1×, postfixed for 20 minutes in 4% paraformaldehide and stored in glycerol at 4°C. Worms were visualized and photographed using an OLYMPUS BX 40 microscope equipped with a SAMSUNG SDC–310 camera connected to a computer running Image Pro software.

### Gene expression analysis

The endogenous expression of *Fh*LAP mRNA (GeneBank AY644459) was determined in NEJs cultured for 48 hours. RNA was extracted from the worms using the RNAqueus-Micro Kit (Ambion, Austin, TX) following the manufacturer's instruction. Any residual DNA remaining in the RNA preparations was removed by DNase digestion using TurboDNase (Ambion). cDNA was synthesized from 500 ng, 50 ng and 5 ng (three 10-fold serial dilutions) of NEJ RNA using the iScript cDNA Synthesis Kit (BioRad, Hercules, CA). *Fh*LAP cDNA was amplified using F: 5′- ATG TGG CCG ATG AGA TTC TGG T-3′; R: 5′-AAT CCA CTA GCC AAT GCC AT-3′, (spanning LAP coding DNA position 1010-1437) amplifying a 427 bp product that span a 3′region from the dsRNA target sequence. *F. hepatica* GAPDH (GenBank AY005475) was used as a control housekeeping gene using the primers F: 5′-GCG CCA ATG TTC GTG TTC GG -3′ ; R: 5′-TGG CCG TGT ACG AAT GCA C -3′ generating a product of 172 bp. *F. hepatica* Cathepsin L3 (*Fh*CathL3) (GenBank DQ534446) was amplified with primers L3F: 5′- TTT CAT ATG AAG CCG AAG GC -3′ and L3R: 5′- GGC TAC TCC AAG CTC TTT CC -3′ producing a 344 bp fragment. *F. hepatica* Cathepsin B2 (*Fh*CathB2) (GenBank DQ534444) was amplified, with primers CB2F: 5′- CAC GGC GGC AGC CAG TG-3′ and CB2R: 5′- TTC GAG AGT CAC CAA CGT GAT C-3′ generating a 250 bp fragment. The presence of the luciferase mRNA was determined by amplification with primers LUCF F: 5′-GTG CCA GAG TCC TTC GAT AG- 3′ and LUCR F: 5′- ACA ACT TTA CCG ACC GCG CC -3′ generating a fragment of 555 bp. The PCR conditions included an initial denaturation at 94°C for 30 sec followed by 35 cycles of 30 sec at 94°C, 60 sec at 55°C for the *Fh*LAP amplicon, 58°C for the GAPDH amplicon, 63°C for the CathL3 and Cath B2 amplicon, and 51°C for LUC amplicon, 60 sec at 72°C and a final extension at 72°C for 10 min. Images of PCR products in ethidium-stained gels were documented using a Versadoc imaging system and QantityOne software (BioRad).

### Enzymatic activity assays

Soluble protein extracts from treated and control juveniles were prepared by sonication-induced lysis (5×5 s bursts on ice, output control value 3, model W-220F Sonicator, Heat Systems—Ultrasonics, Inc., Plainview, New York) in 100 mM glycine, 1 mM MnCl_2,_ pH 8.5. After centrifugation of the lysate for 10 min at 4°C at 14,000 rpm, the supernatant was employed as soluble *F. hepatica* juvenile extract. The protein concentration in the soluble fraction was determined using the bicinchoninic acid assay (BCA kit, Pierce, Rockford, IL). Enzymatic reactions were performed in triplicates using 1 µg of soluble protein and 50 µM of the fluorogenic substrates H-Leu-AMC (Bachem) for LAP or Z-Phe-Arg-AMC (Bachem) for cathepsins L, in a total reaction volume of 200 µl of the appropriate buffer (100 mM glycine, 1 mM MnCl_2,_ pH 8.5 for LAP, and 50 mM sodium phosphate buffer pH 6, 1 mM DTT, 1 mM EDTA for cathepsins). Reactions were incubated at 37°C and the release of AMC was continually monitored at 355 nm (excitation) and 460 nm (emission) for 40 min in a FluoStar Galaxy spectrophotometer (BMG Lab technologies, Toronto).

### Statistical analysis

Student's *t*-test was employed to assess the statistical significance of differences observed. *p* values of the difference are given in the Results section or figure legends.

## Results/Discussion

### Knockdown of luciferase activity in schistosomules

**Figure 1:**
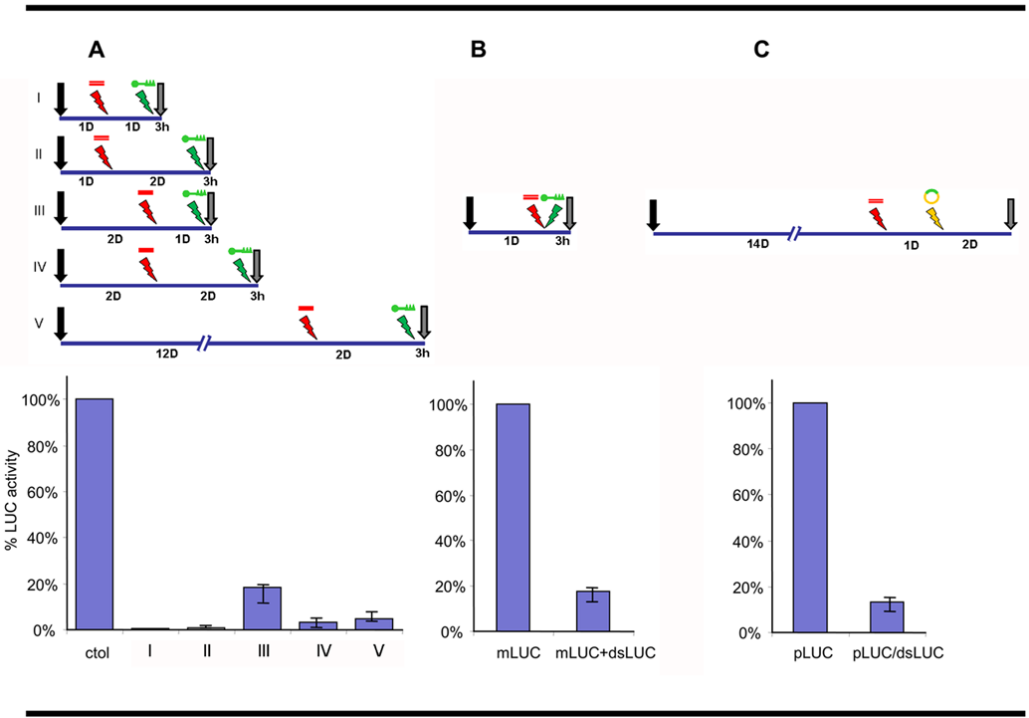
Suppression of exogenous luciferase activity in transfected schistosomules of *Schistosoma mansoni*. Panel A. Five experiments with two sequential electroporations are summarized in the diagram, and the corresponding luciferase activity assays are depicted below. Each experiment consisted of two groups of aproximately 10,000 schistosomules. The treated groups were sequentially electroporated at the indicated times after cercarial transformation (CT, black arrow) whith dsRNA (dsLuc, red) and mRNA (mLuc, green), while the paired control groups were treated only with mLuc at the corresponding time. Three hours after the last electroporation worms were harvested and luciferase activity measured. Activity is expressed as percent of the respective control group that was considered as 100%. For simplicity one bar represents all control groups. Panel B. Relative luciferase activity tested in one day old schistosomules transformed simultaneously with mLuc and dsLuc in a single electroporation. The activity of the control group treated only with mLuc was considered as 100%. Panel C. Relative luciferase activity in fourteen day old schistosomules transformed sequentially with dsLuc and one day later with plasmid encoding firefly luciferase (pLuc, orange), and harvested two days after to measure luciferase activity. Activity is expressed as percent of the control group treated only with pLuc considered as 100%.

In order to establish an informative assay to detect the presence of a viable RNAi pathway, we hypothesized that expression of an exogenous transgene might be inhibited or knocked down by transgene specific dsRNA if the RNAi pathway was present and functional. To validate the system, we tested a protocol using schistosomules of *S. mansoni* as the target helminth because the RNAi pathway is active in the species, and consequently constitutes a positive control for the system. Schistosomules of one, two days or twelve days after mechanical transformations from cercariae were transformed with luciferase dsRNA (dsLuc) by electroporation. At different time points after this treatment with dsLuc, the schistosomules were subjected to a second electroporation in order to introduce luciferase mRNA (mLuc) into their bodies. Three hours after the second electroporation, the transformed schistosomules were harvested and luciferase enzyme activity in their tissues was investigated. As shown in Figure 1A, luciferase activity was strongly reduced in schistosomules electroporated with dsLuc following introduction of mLuc. By contrast, luciferase activity was readily apparent in control schistosomules transformed with mLuc only. Specifically, there was a significant reduction in the luciferase activity in all treated groups of >95% in one day old schistosomules (p<0.05) and >85% in two day old schistosomules (p<0.05). The strong RNAi effect was still detectable in schistosomules treated 12 days after cercarial transformation. The period between the dsRNA transfection and the mRNA transfection (one or two days) showed small, non-significant variations in the levels of knockdown of luciferase activity.

Next, we investigated the effect of the co-transfection by electroporation of one day old schistosomules with dsLuc and Luc mRNA simultaneously. In similar fashion, we observed robust silencing, specifically 82% reduction in the luciferase activity (p<0.05) (Figure 1B). Finally, we transformed fourteen day old schistosomules with dsLuc, and one day later transfected them by electroporation with a plasmid DNA construct encoding luciferase driven by the actin 1.1 gene promoter of *S. mansoni* (pLuc) (from ref [14]). We selected to transform two week old schistosomules because it was reported that actin expression increased dramatically by that time [47]. Consistently when luciferase activity was measured two days after the last electroporation, the control group transformed with pLuc only displayed strong levels of activity, indicating that the plasmid was able to drive the *in vivo* expression of the transgene. However, the dsLuc treated group showed a reduction of more than 80% in luciferase activity (p<0.05).

Taken together these data indicate that dsRNA is able to induce a strong silencing response of a reporter mRNA in schistosomules irrespectively of the exogenous (electroporated) or endogenous (expressed from plasmid) origin of the transcript. Furthermore, these results indicate that we have developed a quick and efficient system to detect an active RNAi pathway that can be applied to evaluate the viability of RNAi in other parasites.

### The reporter protein firefly luciferase is active in juveniles of *F. hepatica*

**Figure 2:**
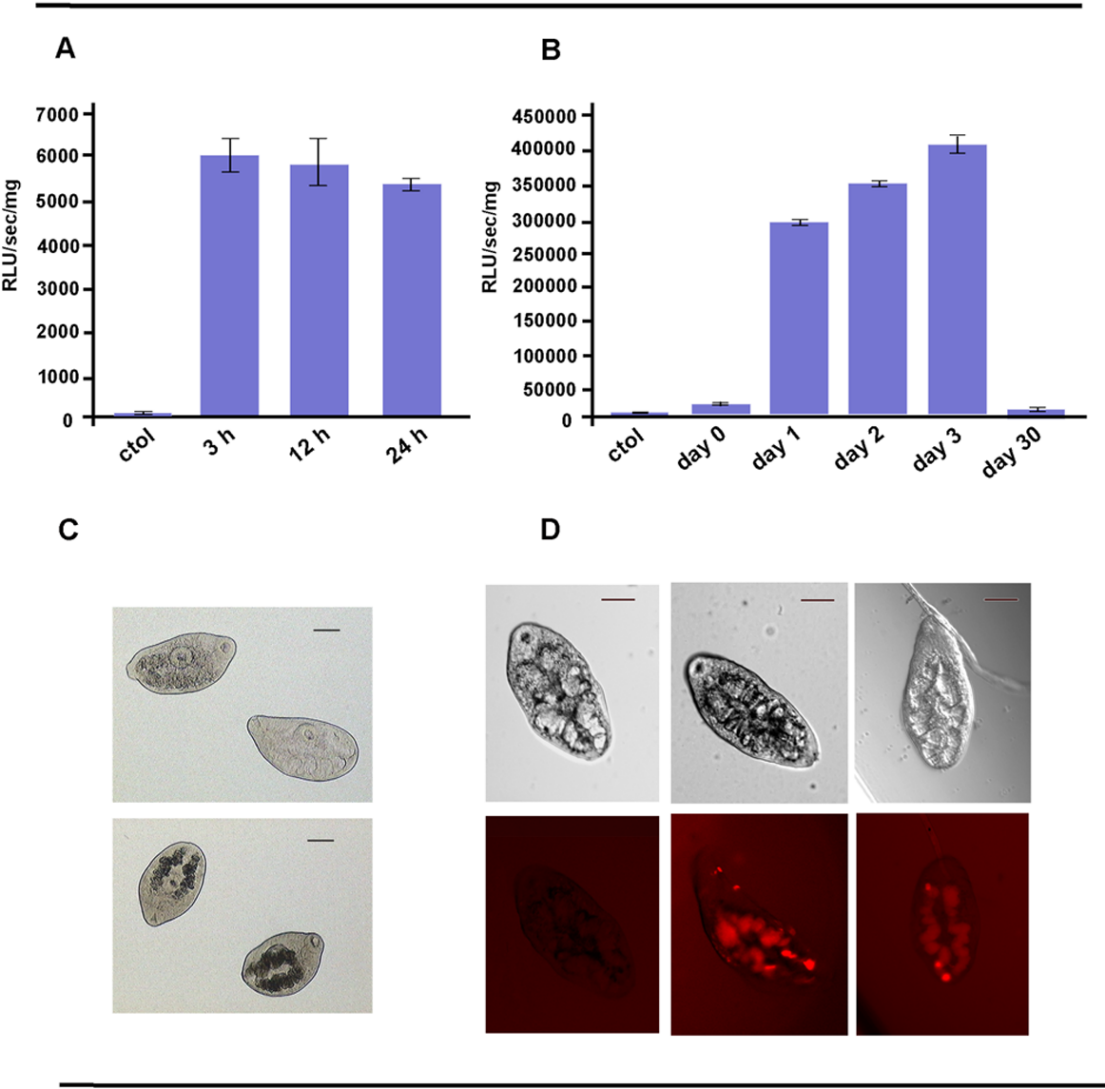
Luciferase activity in transfected *Fasciola hepatica* newly excysted juveniles. Panel A. Detection of luciferase activity at the indicated times post-electroporation of the mLuc. Differences between the mLuc treated groups and non treated group (control) were significant (p<0.05). Panel B. Juvenile worms at the indicated times post excystement were electroporated with 50 ng/µl of luciferase mRNA (mLuc), incubated for three hours, and the luciferase activity in their tissue was measured. A mock electroporation, control group was included (electroporated in the absence of exogenous mRNA). Difference between 1, 2 or 3 day old NEJs and the control group are strongly significant (p<0.01). Panel C. Localization of mLuc in juvenile worms. Two day old NEJs were mock electroporated or with 50 ng/µl of mLuc, fixed three hours later and processed for whole mount hybridization. Digoxigenin labeled antisense probe detected mLuc in the digestive tract of treated worms (lower panel) while no signal was detected in mock electroporated worms (upper panel). Scale bar represent 50 microns. Panel D. Two day old juvenile worms electroporated with 0, 50 or 100 ng/µl of Silencer Cy3 fluorescent siRNA, and followed by fluorescent microscopy. Representative images of live worms maintained in culture for 24 hours after treatment are shown, with normal field in the upper panels and fluorescence in the lower panels.

We anticipated that the test system described above, knockdown of exogenous firefly luciferase activity with specific dsRNA, would facilitate determination of the presence of a functional RNAi pathway in less well studied species, and we selected *F. hepatica* to test this hypothesis. As a first component in the development of a tractable system for RNAi in less well studied flukes, we tested electroporation since it is the most proficient method for delivery of dsRNA in schistosomules of *S. mansoni* [20]. One day old juveniles of *F. hepatica* were transformed with 50 ng/µl mLuc by square wave electroporation (a single 20 millisecond pulse of 125 volts), and three hours later were snap frozen at −80°C for subsequent analysis of luciferase activity. Significant amounts of luciferase activity (>300,000 RLUs/sec/mg) were detected in homogenates of the transformed flukes while untreated worms expectedly show neglectable activity (p<0.001). (see Figure 2B, day 1). No obvious detrimental effects due to the electroporation were observed, and within few minutes of the electroporation the worms recovered normal vitality and motility.

Since mRNAs usually exhibit short half lives *in vivo*, we also examined luciferase activity at various times post-electroporation in 2 days old treated juveniles, as an indirect measure of mLuc stability. As shown in Figure 2A, the luciferase levels peaked in our study time points at 3 h post electroporation (p<0.05), and had begun to decline by 12 h and 24 h.

We also examined if there were variations in succeptibility to electroporation, by perfoming the procedure at different times after excystment of metacercariae. NEJs (1 hour old) or cultured larvae of *F. hepatica* one, two, three and 30 days old were transformed with 50 ng/µl mLuc by square wave electroporation (a single 20 millisecond pulse of 125 volts), and three hours later were snap frozen at −80°C for subsequent analysis of luciferase activity. Figure 2B presents the luciferase activity in tissues of the transformed flukes measured at three hours after electroporation. Optimal luciferase activity was seen in three-day-old juveniles (>400,000 RLUs/sec/mg), with elevated levels also detected in one- and two-day-old juveniles (p<0.01). Much lower, though nonetheless substantial, levels of activity were detected in the one-hour-old NEJs (∼25,000 RLUs/sec/mg) and the 30-day-old juveniles (∼18,000 RLUs/sec/mg). The elevated levels in the younger flukes compared to the 30-day-old juveniles may reflect developmental differences in the translation of the mLuc or, trivially, may reflect decreased vitality in the larval flukes as the consequence of extended culture *in vitro*. Long term culture of larval *F. hepatica* is known to be challenging [48].

Collectively, these findings demonstrated that square wave electroporation could deliver efficiently exogenous nucleic acids into the tissues of cultured juvenile *F. hepatica*. Given the apparent success with these electroporation settings, we employed similar conditions in the experiments described below - NEJs of *F. hepatica*, 50 ng/µl of RNA, a single square pulse of 20 msec at 125V, and analysis of firefly luciferase activity three hours post-electroporation.

The intra-parasite localization of the transfected molecule was investigated by electroporating two days old NEJs with mLuc, and detecting the presence of the mRNA by whole mount (*in toto*) hybridization 3 hours later. The antisense labeled probe produce a strong signal in the digestive tract of the treated worms, while no signal was detected in mock electroporated control worms (Figure 2C). To confirm these results we electroporated two days old NEJs with a labeled siRNA used as transfection control in siRNA experiments in mammals. Three hours after electroporation treated worms showed a pale fluorescent signal in their parenchyma and a clear uptake in the digestive ceca, while no labeling was detected in the control worms (Figure S1). The signal persisted after 24 hs in live worms as depicted in Figure 2D. These data suggest that the digestive tract of the worm might be the principal site of uptake of the transfected molecules, although other entry pathways cannot be ruled out. A similar picture was obtained in sever day old *S. mansoni* schistosomules [20]. However, in this study the fluorescence pattern seen in the day 7 cultured schistosomes was distinct from that seen following electroporation of fresh (0 day) parasites. Similarly, other reports indicate that the main targets of electroporation in *S. mansoni* and *S. japonicum* are tegumental and subtegumental cells [12],[13]. The differences within schistosomes have been related to different maturation status, since it has been reported that schistosomula mouth remains closed until around day 7 after transformation [20]. In *F. hepatica*, the metacercarial gut is filled with secretory vesicles that are released during excystement, and the digestive tract of the juvenile remains a major source of secretions [49]. Consequently the digestive tract of the juveniles remains open from excystment on, and therefore this could be the entry path of the electroporated molecules, as indicated by the present data. Moreover, this suggests that electroporation could be an appropriate delivery method to knock down specific genes expressed in digestive tract.

### RNAi pathway functions in *F. hepatica* juveniles

**Figure 3:**
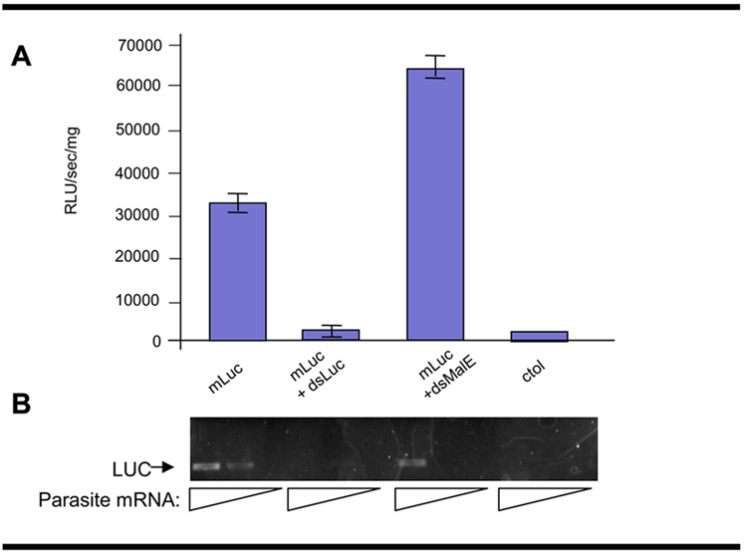
Suppression of exogenous luciferase activity in transformed *F. hepatica* newly excysted juveniles (NEJ). Panel A. One day old NEJ were transformed by electroporation with dsLuc or an irrelevant dsRNA, dsMalE. One day later, they were again transformed by electroporation, this time with mLuc. Untreated worms indicate the absence of luciferase activity in *F. hepatica* NEJ. There was a significant reduction in the luciferase activity in worms treated with dsLuc+mLuc (*p*<0.05). Unexpectedly, the activity was higher in the worms treated with the irrelevant dsMalE than in the control treated only with mLuc. Panel B. mLuc amplified by RT-PCR from serial dilutions of total RNA obtained on specimens fixed in formalin 48 hours after the electroporation.

Because we observed that *F. hepatica* NEJ and juveniles could be productively transformed with mRNA by square wave electroporation, and because reporter firefly luciferase was active in these flukes, we proceeded to attempt to silence the expression of the exogenous reporter. In particular, one-day-old juveniles were transformed by electroporation with either dsLuc or dsMalE, the latter representing an irrelevant control dsRNA. Twenty fours hours later, these transformed flukes were subjected to a second electroporation procedure involving transformation with mLuc. A third group of flukes was electroporated with mLuc only, i.e. treatment with dsRNA, while a fourth group was not treated with either dsRNA or mLuc. As shown in Figure 3A, at 3 h after electroporation of mLuc, luciferase activity of ∼35,000 RLU/sec/mg was recorded in flukes treated only with mLuc. As expected, negligible luciferase activity was detected in the tissues of control flukes not exposed to either dsRNA or mRNA. By contrast, the luciferase activity in the juvenile flukes exposed to both dsLuc and mLuc was almost completed ablated (2000 RLUs/sec/mg; >95% reduction, p<0.001). Unexpectedly, the juvenile worms treated with the irrelevant MalE dsRNA showed an increase in luciferase activity. We found no reasonable explanation for this observation.

The direct measurement of luciferase activity by luminometry provides a demonstration of the gene silencing at a protein level. In addition to this, we employed RT-PCR to investigate the effect at mRNA levels. The mLuc was present at similar levels in the no dsRNA and the irrelevant control (dsMalE) groups, but substantially reduced in the flukes treated with dsLuc, indicating that the effect was specific (Figure 3B). The findings demonstrated that a viable and efficient RNAi pathway existed in juveniles of *F. hepatica*. In this regard, they confirm the findings of McGonigle et al. (2007) who recently reported RNAi knock-down of the papain-like cysteine proteases cathepsin L and cathepsin B in juvenile *F. hepatica* by soaking [35]. Our electroporation protocol produces a detectable effect at the RNA level using less dsRNA than the soaking protocol used by McGonigle *et al*. However, since neither of these studies analyzed the required amount of dsRNA required for getting a detectable effect, is still not possible to determine the more efficient delivery method. In schistosomes comparative studies indicate that electroporation outperforms soaking as a delivery method [20], a fact that still need to be addressed in *Fasciola* and other species and models.

### Suppression of an endogenous gene in juveniles of *F. hepatica*

**Figure 4:**
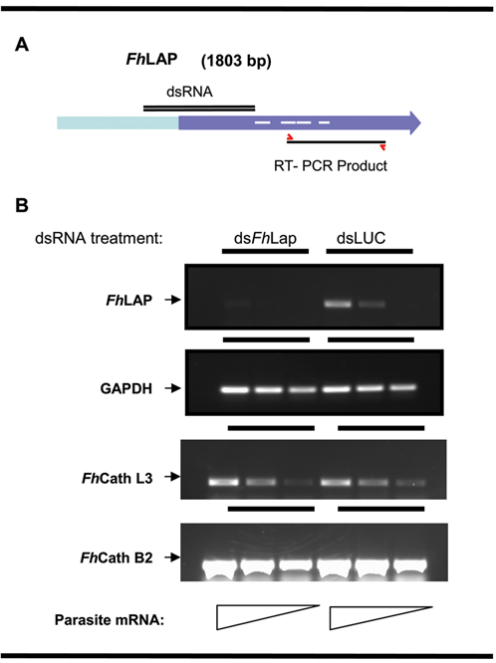
RNA interference of the endogenous *Fasciola hepatica* leucine aminopeptidase (*Fh*LAP). Panel A. Schematic representation of the *Fh*LAP gene, and the regions targeted by the dsRNA and amplified by PCR to test the effect. The region encoding the conserved carboxy domain of LAP is indicated by darker shading, with the signature active site regions indicated by white lines. Panel B. RT-PCR products amplified from serial dilutions of total RNA obtained 48 hours after the electroporation of NEJ with dsLap or dsLuc as an irrelevant dsRNA control. Reductions of mRNA were established by comparison to the amplification of the conserved GAPDH, and the juvenile specific CathB2 and CathL3 genes.

Having demonstrated the existence of a viable RNAi pathway in juveniles of *F. hepatica* through the knockdown of the exogenous reporter, firefly luciferase, we proceeded to investigate RNAi in this species by targeting an endogenous *F. hepatica* gene, and we selected leucine aminopeptidase as an interesting enzyme [46]. We electroporated 12 day-old-juveniles with gene specific dsRNA of F. *hepatica* leucine aminopeptidase (*Fh*Lap), and dsLuc as an irrelevant control. No phenotypic effects were observed by light microscopy for these flukes maintained for 48 hours after electroporation (not shown) at which time the tissues of the worms were harvested. We could not detect LAP enzymatic activity in somatic extracts of both treated and control worms. However, the activity of the highly expressed cathepsin Ls was detectable at very low levels in both samples, suggesting that we might be under the limit of detection for LAP (data not shown). Total RNA was extracted and employed as template for semi-quantitative RT-PCR analysis. The primers utilized targeted a region discrete from the target RNAi locus in the *Fh*Lap cDNA (Figure 4, panel A). This strategy was employed to obviate residual dsRNAs serving as spurious template for the RT-PCRs [50]. Thus the dsRNA spans residues 389–871 of the cDNA while the RT-PCR targets residues 1010–1437. As shown in Figure 4B, there was a potent knock-down of mRNA encoding *Fh*Lap in the worms treated with ds*Fh*Lap but not in those treated with dsLuc. Moreover, no differences between groups of parasites were detected for the amplification of the housekeeping GAPDH gene (internal control) or the cathepsins L3 and B2, predominantly expressed in juveniles [51]. The reduction of the mRNA levels of *Fh*LAP, and the absence of variations in three other *F. hepatica* genes indicated that the silencing was specific for the target *Fh*Lap gene, excluding off-targeting effects. These findings demonstrated RNAi silencing of an endogenous gene at mRNA level, following on from our initial demonstration of knock-down of the exogenous reporter gene encoding firefly luciferase at mRNA and also at protein level.

### Concluding remarks

Because we are interested to transfer reverse genetics tools developed for *Schistosoma* to less well studied flukes or other helminths, we designed a reporter system for RNAi using an exogenous gene, and tested it in the liver fluke *Fasciola hepatica*. We demonstrated both the applicability of the system and consequently confirmed the existence of a viable RNAi pathway in this parasite. This straight-forward reporter system could provide investigators with a tool to test the presence of a functional RNAi pathway in other parasites that are by other means intractable.

It is noteworthy that despite the success of the RNAi in *Caenorhabditis elegans,* the technique has serious limitations in other nematodes, both free living and parasitic [52]. Viney and Thompson recently postulated that either the delivery methods applied in parasitic nematodes were inapropiate, or they are defective for genes required to initate RNAi from external dsRNA [53]. While soaking is the delivery method most frequent chosen when testing for the presence of RNAi, some genes relevant for the uptake and spreading of the silencing phenomena might be missing. In effect, in *Caenorhabditis briggsae* the absence of the SID-2 gene is responsible for the failure to induce RNAi externally (by soaking or feeding) while is effective when microinjected or electroporated [54]. Furthermore, the draft genome of the filarial nematode *Brugia malayi* reveals the absence of sid-1 and sid-2 genes, indicating that spreading of RNAi would not occur in this parasite [55]. The procedure here described could provide a rapid and inexpensive method to help decide if this is the case in other parasitic helminths.

In conclusion, the present investigation achieved three goals. First, using *S. mansoni* as model, we developed an efficient and quick system to test the presence and viability of an intact RNAi pathway in parasites in which the RNAi has no been tested yet and/or the long term culture conditions are not yet established. Second, we introduced genetic material by electroporation into *F. hepatica* demonstrating the feasibility of this route of transformation of this trematode. Third, we demonstrated the existence of a viable and functional RNAi pathway in *F. hepatica* by knocking down a reporter gene, and an endogenous gene, establishing the starting point for functional genomic studies in *Fasciola*. We consider that these findings will not only enhance investigation of gene function in *Fasciola*, including investigation of novel intervention targets, but they may likewise provide a path forward for genetic manipulation of even less studied trematodes.

## Supporting Information

Figure S1 Labeled siRNA detection in live *Fasciola hepatica* newly excysted juveniles. Panel A. Two day old juvenile worms were electroporated with 0, 50 and 100 ng/μl of a Cy3 labeled control siRNA, and maintained in culture in Transwell inserts (Costar). After 3 hs they were visualized in a fluorescent microscope. Representative images of live worms are shown, with normal field at the left and fluorescence at the right, and the previously indicated increasing concentrations from top to bottom. The dotted appeareance of the clear field is due to the Transwell membrane. Panel B. A topographic image of parasites after 3 hs of being electroporated with 50 ng/μl of the labeled siRNA removed from the Transwell insert. While most of the label is in the digestive ceca, a diffuse labeling in some worms is detected. Panel C. Persistence of the fluorescent signal after 24 hs of culture observed in live worms. From left to right 0, 50 and 100 ng/μl treated worms, with clear field on top and fluorescent images below. Scale bars represent 50 microns.
(5.89 MB TIF)

Alternative Language Abstract S1 Translation of the Abstract into Spanish by José F. Tort
(0.01 MB PDF)
