# Supplementary figures and images for: Development of Functional Genomic Tools in Trematodes: RNA Interference and Luciferase Reporter Gene Activity in Fasciola hepatica
Source: PLoS Negl Trop Dis. 2008 Jul 9;2(7):e260. doi: 10.1371/journal.pntd.0000260 (PMC2440534; doi:10.1371/journal.pntd.0000260)

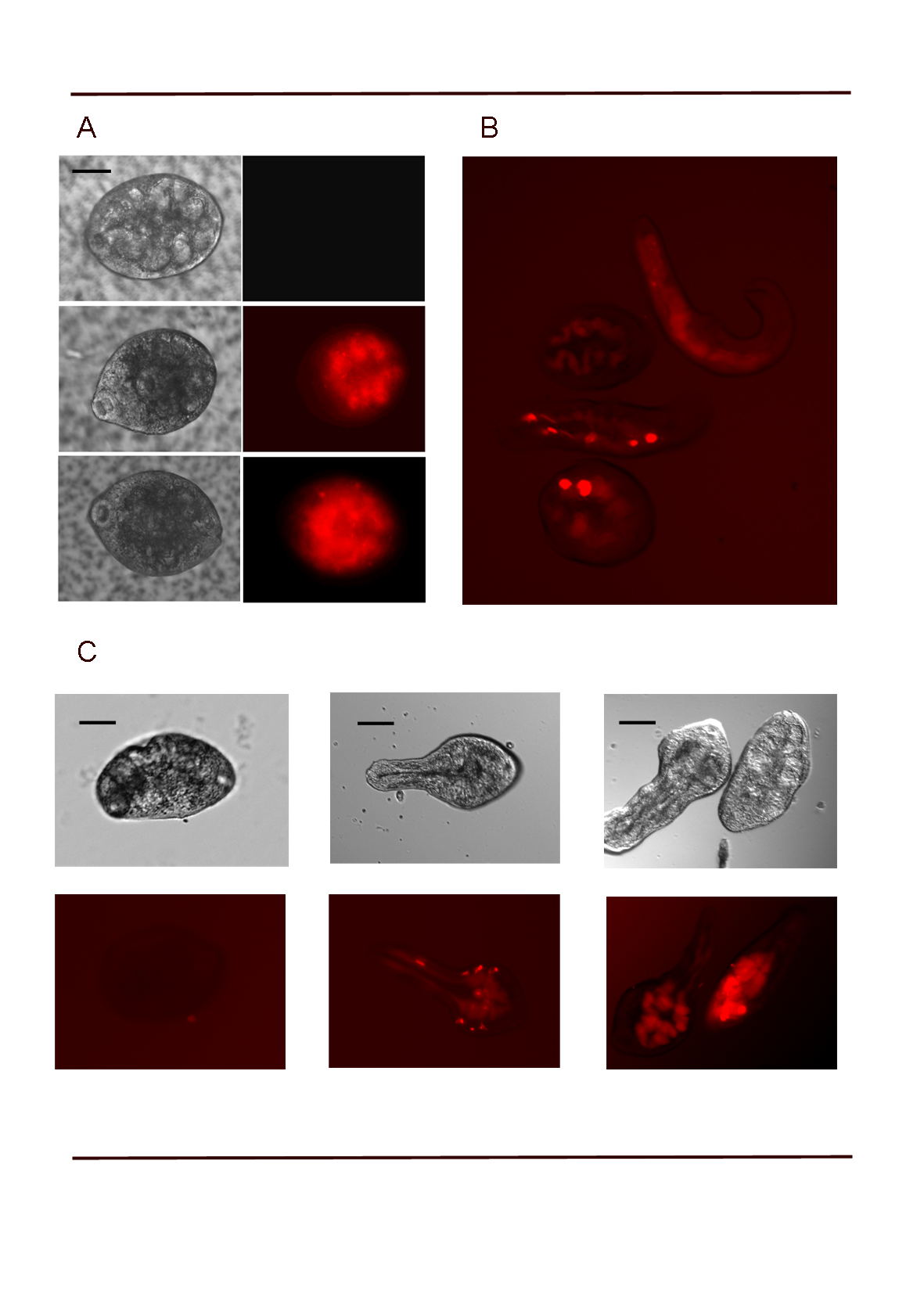

Supplement: Figure S1 — Labeled siRNA detection in live Fasciola hepatica newly excysted juveniles. Panel A. Two day old juvenile worms were electroporated with 0, 50 and 100 ng/μl of a Cy3 labeled control siRNA, and maintained in culture in Transwell inserts (Costar). After 3 hs they were visualized in a fluorescent microscope. Representative images of live worms are shown, with normal field at the left and fluorescence at the right, and the previously indicated increasing concentrations from top to bottom. The dotted appeareance of the clear field is due to the Transwell membrane. Panel B. A topographic image of parasites after 3 hs of being electroporated with 50 ng/μl of the labeled siRNA removed from the Transwell insert. While most of the label is in the digestive ceca, a diffuse labeling in some worms is detected. Panel C. Persistence of the fluorescent signal after 24 hs of culture observed in live worms. From left to right 0, 50 and 100 ng/μl treated worms, with clear field on top and fluorescent images below. Scale bars represent 50 microns. (5.89 MB TIF) [file pntd.0000260.s001.tif]
