# Supplementary material for: Development of Functional Genomic Tools in Trematodes: RNA Interference and Luciferase Reporter Gene Activity in Fasciola hepatica
Source: PLoS Negl Trop Dis. 2008 Jul 9;2(7):e260. doi: 10.1371/journal.pntd.0000260 (PMC2440534; doi:10.1371/journal.pntd.0000260)
Supplement: Alternative Language Abstract S1 — Translation of the Abstract into Spanish by José F. Tort (0.01 MB PDF) [file pntd.0000260.s002.pdf]

Desarrollo de herramientas de genómica funcional en tremátodos: Interferencia de ARN y actividad reportera de luciferasa en *Fasciola hepatica*

Gabriel Rinaldi [1, 2], Maria E. Morales [1], Martín Cancela [2], Estela Castillo [3], Paul J. Brindley [1 +], and José F. Tort [2 *]

Resumen

ANTECEDENTES

La creciente disponibilidad (a partir de proyectos genoma y transcriptoma) de información de secuencias de diversos parásitos, ofrece nuevas oportunidades para la identificación de mediadores clave en la interacción huésped-parasito. Las herramientas de genómica funcional y los métodos de manipulación de genes son esenciales para descifrar el papel de estos genes e identificar nuevos blancos de intervención antiparasitaria. Esperanzadores avances en la genómica funcional de helmintos parásitos comienzan a surgir como la expresión de transgenes y la interferencia de ARN (ARNi) reportada en varias especies de nemátodos, pero el área está aun en pañales en platelmintos, con reportes en apenas tres especies. Mientras se avanza en los organismos modelo, existe la necesidad de extender rápidamente estas tecnologías a otros parásitos responsables de varias enfermedades crónicas en humanos y en ganado.

METODOLOGIA, DESCUBRIMIENTOS PRINCIPALES

Como forma de extender estos enfoques a helmintos parásitos menos estudiados, desarrollamos un método para testar la presencia de una vía funcional de ARNi silenciando un gen reportero exógeno, la luciferasa de la luciérnaga (fLUC). Establecimos el método en el parásito humano *Schistosoma mansoni* y confirmamos su utilidad en la duela del hígado *Fasciola hepatica*. Transformamos juveniles recientemente desenquistados de *F. hepatica* por electroporación con ARN mensajero de fLUC y pudimos detectar luego de tres horas, actividad luciferasa, la que aparece asociada principalmente a los ciegos digestivos. A continuación, investigamos la presencia de una vía de ARNi activa en *F. hepatica* haciendo el knock down de la actividad exógena de luciferasa mediante la introducción en los parásitos transformados de ARN doble cadena (ARNdc) específico para fLUC. Además confirmamos la vía de interferencia tomando como blanco un gen endógeno de *F. hepatica* codificante para la leucin-aminopeptidasa (*Fh*LAP), observando una reducción significativa en los niveles del ARNm específico.

CONCLUSIONES/SIGNIFICANCIA

En resumen, estos estudios demuestran la utilidad del ARNi dirigido al gen reportero fLUC como un ensayo indicador sencillo para establecer la presencia de una vía intacta de ARNi en helmintos parásitos. Esto puede facilitar el estudio de la función génica y la identificación de blancos relevantes para la intervención en organismos hasta ahora intocados por estas técnicas. Mas específicamente, estos resultados abren nuevas perspectivas para la genómica funcional en *F. hepatica*, que podrían conducir al desarrollo de nuevos mecanismos de intervención contra la fascioliasis.
